# Syndrome de Naffziger

**DOI:** 10.11604/pamj.2014.18.281.5198

**Published:** 2014-08-06

**Authors:** Issam Elouakili, Younes Ouchrif

**Affiliations:** 1 Service de Chirurgie Orthopédique, CHU de Rabat, Maroc

**Keywords:** Syndrome de Naffziger, cote surnuméraire, malformation, Naffziger syndrome, supernumerary rib, malformation

## Image en medicine

Le syndrome de Naffziger ou le syndrome de la côte cervicale est un ensemble de symptômes causés par une maladie relativement rare de malformation congénitale osseuse. Ce syndrome est également appelé ou syndrome du scalène antérieur. Ce syndrome ce caractérise par l'existence d'une côte cervicale supplémentaire. Cette côte e naît à la septième vertèbre cervicale. Il s'agit d'une anomalie congénitale située au-dessus de la première côte normale. Cette anomalie congénitale, peut engendrer une compression du plexus brachial ou des éléments vasculaire au niveau cervicale. Nous rapportant le cas d'un patient de 40 ans, qui présente des signes d'irritation du plexus brachial, et dont les examens complémentaires ont objectivé une image de cote surnuméraire.

**Figure 1:**
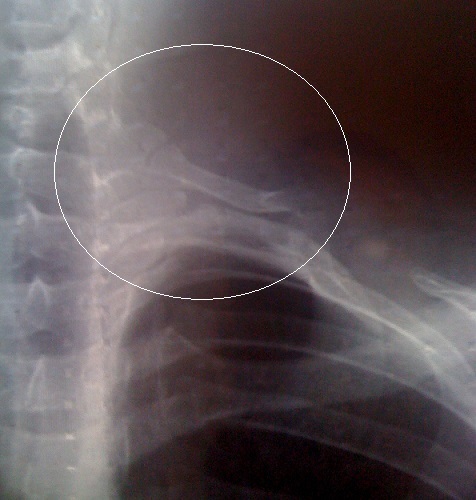
Radiographie du thorax montre la cote surnuméraire

